# Psychometric Validation of the Italian 8‐Item Green Paranoid Thoughts Scale (GPTS‐8)

**DOI:** 10.1002/mpr.70097

**Published:** 2026-09-30

**Authors:** Antonio Preti, Carmelo Masala, Donatella Rita Petretto

**Affiliations:** ^1^ Dipartimento di Neuroscienze “Rita Levi Montalcini” Universita Degli Studi di Torino Turin Piedmont Italy; ^2^ Dipartimento di Pedagogia Psicologia Filosofia Universita Degli Studi di Cagliari Cagliari Sardinia Italy

**Keywords:** bifactor analysis, confirmatory factor analysis, paranoia, persecution, self‐assessment

## Abstract

**Background:**

Paranoid ideation ranges from mild social‐evaluative concerns to persecutory beliefs. The eight‐item Green Paranoid Thoughts Scale (GPTS‐8) provides a brief assessment of this continuum through reference and persecution items.

**Aims:**

To examine the psychometric properties of the Italian version of the GPTS‐8, including its factorial structure, reliability, and convergent validity, in a non‐clinical university sample.

**Methods:**

243 university students completed the Italian GPTS‐8 and the Prodromal Questionnaire–Brief (PQ‐B). Confirmatory factor analyses compared unidimensional, two‐factor, bifactor, and second‐order models using maximum likelihood estimation with robust standard errors. Reliability was evaluated using Cronbach's *α* and McDonald's ω, and item‐level contributions to the general factor were examined using bifactor indices (ECV, ωH, IECV).

**Results:**

The bifactor model showed the best fit, supporting a dominant general paranoia factor alongside specific reference and persecution dimensions. Internal consistency was good for the GPTS‐8 total score (*α* = 0.78) and acceptable for the subscales (respectively, 0.68 and 0.66). Item‐level diagnostics indicated that several items primarily reflected the general factor, consistent with a hierarchical organization of paranoid ideation. Item response patterns suggested greater sensitivity at moderate to higher levels of the latent trait. Participants scoring above established PQ‐B thresholds showed significantly higher GPTS‐8 scores across dimensions, supporting convergent validity.

**Conclusions:**

The Italian GPTS‐8 showed satisfactory psychometric properties, providing incremental evidence for its structural validity in an Italian‐speaking context. However, given the use of a convenience student sample, findings should be considered preliminary and not directly generalizable to broader or clinical populations.

## 1 Background

Paranoid ideation is increasingly recognized as a transdiagnostic phenomenon that extends beyond psychotic disorders and is distributed along a continuum of severity in the general population (Freeman et al. 2005; Neidhart et al. 2024). Contemporary cognitive and epidemiological models conceptualize paranoia as a hierarchically organized construct, in which relatively common ideas of social reference occupy the lower end of the distribution, while persecutory beliefs represent more severe and clinically consequential expressions. This dimensional architecture highlights the need for measurement instruments capable of capturing individual differences across the full range of paranoid ideation.

The Green Paranoid Thoughts Scale (GPTS; Green et al. 2008) and its revised version, the Revised Green Paranoid Thoughts Scale (R‐GPTS; Freeman et al. 2021), are widely used tools for operationalizing this continuum. Their strong psychometric properties and applicability across clinical and non‐clinical populations are well established (Statham et al. 2019; Latteur et al. 2022; Schlier et al. 2024). Both instruments distinguish between ideas of reference—beliefs that neutral events or others' behaviors carry personal significance—and ideas of persecution, which capture the belief that others intend harm. Empirical evidence consistently supports a hierarchical organization in which reference ideas are relatively prevalent in the general population, whereas persecutory beliefs emerge primarily at higher levels of severity (Freeman et al. 2005; Neidhart et al. 2024).

Despite their strengths, the length of the original GPTS and the multidimensionality of the reference subscale limit feasibility in large‐scale surveys, digital assessments, and studies requiring repeated measurement, where brevity and psychometric transparency are essential. To address these constraints, Bianchi and Verkuilen (2021) developed the GPTS‐8, an eight‐item short form that retains the core reference–persecution framework while substantially reducing respondent burden. The scale was derived through empirically and theoretically guided item reduction aimed at improving factorial validity and minimizing redundancy in the longer GPTS versions. By retaining four ideas‐of‐reference items and four ideas‐of‐persecution items, the brief form preserves balanced coverage of the original domains while exhibiting a cleaner factor structure and external associations comparable to those of the longer versions. Subsequent validation studies in French‐ and Arabic‐speaking samples further demonstrated good reliability, structural coherence, and promising cross‐cultural consistency (Raffard et al. 2023; Fekih‐Romdhane et al. 2025).

To date, however, no validated Italian version of the GPTS‐8 is available. This gap limits the use of a brief paranoia measure in one of Europe's largest linguistic communities and constrains cross‐national comparability in epidemiological and psychometric research. An Italian adaptation of the GPTS‐8 would facilitate harmonization with emerging international datasets and support research adopting dimensional and latent variable models of paranoia.

The present study provides the first validation of the GPTS‐8 in Italian. Specifically, we examined its factorial structure, internal consistency, and convergent validity in a non‐clinical sample of young adults. Consistent with contemporary hierarchical models of paranoia, we compared several plausible structural representations of the GPTS‐8, including unidimensional, correlated‐factor, second‐order, and bifactor models. The bifactor approach allows item variance to be decomposed into a general paranoia factor and specific ideas‐of‐reference and persecution factors, thereby clarifying whether the subscales contribute meaningful variance beyond a dominant general dimension (Reise 2012; Rodriguez et al. 2016a, 2016b). This approach was also adopted in the original development of the GPTS‐8 (Bianchi and Verkuilen 2021). By integrating model comparisons and item‐level diagnostics, this study aims to clarify the latent structure of the GPTS‐8 and provide a methodological foundation for its use in Italian psychometric and cross‐cultural research.

## 2 Methods

The study was conducted at a public university located in a major city in Sardinia, Italy. The institution offers undergraduate and postgraduate programs across a broad range of disciplines, including Medicine, Law, Engineering, Philosophy, and Natural Sciences.The protocol received institutional review board approval and adhered to the Declaration of Helsinki and later amendments (Resneck 2025).

All participants provided written informed consent and participated voluntarily without compensation.

### 2.1 Participants

Consistent with the original validation strategy of the instrument (Bianchi and Verkuilen 2021), we recruited a non‐clinical sample of students. Undergraduate students were recruited using a snowball (chain‐referral) sampling procedure initiated across five academic areas (Medicine, Law, Engineering, Philosophy, and Natural Sciences) to enhance disciplinary dispersion. Participants completed the questionnaire booklet and subsequently referred other eligible peers. This network‐based, non‐probability strategy was adopted to increase within‐university heterogeneity and reduce single‐course clustering. Snowball sampling is appropriate for examining internal structure and reliability; however, it does not ensure population representativeness and may introduce homophily‐related selection bias.

The final sample comprised 243 students (109 men, 134 women) aged 19–34 years (*M* = 24.3, SD = 3.5, Median = 24), with no significant gender differences in age. The sample size was considered adequate for evaluating the factorial structure and convergent validity of the GPTS‐8, given the limited number of observed variables (8 items), the high participant‐to‐item ratio (> 30:1), the moderate complexity of the tested models, and evidence suggesting that sample‐size adequacy in structural equation modeling depends primarily on model characteristics rather than universal numerical thresholds (Wolf et al. 2013).

### 2.2 Assessment

The Green Paranoid Thoughts Scale – eight‐item version (GPTS‐8; Bianchi and Verkuilen 2021) is a short form of the R‐GPTS (Freeman et al. 2021). It includes four items on ideas of social reference (e.g., “I often heard people referring to me”) and four on persecution (e.g., “People have been hostile toward me on purpose”). Each item is rated on a five‐point Likert‐type scale ranging from 1 (“Not at all”) to 5 (“Totally”), referring to experiences in the past month. Scores can be computed for total paranoia (8–40) and for each subscale (4–20). The Italian adaptation of the original 32‐item GPTS showed good reliability and convergent validity in a non‐clinical sample (Preti et al. 2019).

The translation and cultural adaptation of the GPTS‐8 into Italian followed a standard forward–backward procedure. The English version reported in the original validation studies (Bianchi and Verkuilen 2021; Raffard et al. 2023) was translated into Italian by a native Italian psychiatrist fluent in English. The translated items were compared with an existing Italian version of the original GPTS from which the eight items were derived (Preti et al. 2019) to ensure terminological consistency.

A second Italian psychiatrist, fluent in French, reviewed the Italian translation against the published French version of the GPTS‐8 (Bianchi and Verkuilen 2021) and provided recommendations for linguistic refinement. An independent psychologist fluent in English then performed a back‐translation into English. This version was reviewed against the original English scale by a further bilingual psychologist, and minor revisions were made to achieve semantic equivalence. Readability was assessed using the GULPEASE index (Lucisano and Piemontese 1988), yielding a score of 73, indicating good comprehensibility for individuals with lower secondary education and very good comprehensibility for those with upper secondary education. Finally, face validity was evaluated in a pilot test involving five undergraduate students not included in the study sample. Participants assessed item clarity, identified ambiguous wording, and evaluated ease of completion. No substantive issues emerged, and the finalized version was retained for the study.

The Prodromal Questionnaire–Brief (PQ‐B; Loewy et al. 2011; Pelizza et al. 2018) assesses positive psychotic‐like symptoms. It comprises 21 yes/no items with distress ratings for endorsed symptoms. Scoring includes the number of endorsed items, distressing items, and total distress score. In Italian samples, cut‐offs of 7 (frequency) and 20/22 (distress) identify individuals at higher risk for psychosis (Pelizza et al. 2018; Preti et al. 2018). Fifty‐four participants (22%) scored above the PQ‐B frequency cut‐off, 49 (20%) above the distress cut‐off, and 45 (18%) above both, forming the at‐risk subgroup.

### 2.3 Data Analysis

Statistical analyses were conducted using the *Statistical Package for the Social Sciences* (SPSS, version 29; IBM Corp., 2023) and dedicated packages in *R* (version 4.3.2; R Core Team 2023). All tests were two‐sided, and a two‐tailed *α* level of 0.05 was considered statistically significant.

Categorical variables were summarized as counts and percentages, while continuous variables were described using means and standard deviations. Skewness and kurtosis were examined to assess univariate normality, applying conservative thresholds of skewness < 2 and kurtosis < 7 (Hair et al. 2010). Group comparisons were conducted using Student's *t*‐test or Welch's *t*‐test when the assumption of homogeneity of variance was violated. Effect sizes for differences in continuous variables were estimated using Hedges' *g*, interpreted according to conventional benchmarks (Cohen 1988): approximately 0.20 (small), 0.50 (medium), and ≥ 0.80 (large). Correlations were assessed with Pearson's *r* and interpreted as small (≈ 0.10), medium (≈ 0.30), or large (≥ 0.50) effects (Cohen 1988).

Reliability was assessed in terms of internal consistency using Cronbach's *α*. Values above 0.60 were considered indicative of moderate reliability and those above 0.70 of good reliability (Taber 2018). For multidimensional tools, subscale reliability coefficients above 0.60 are considered acceptable, especially when subscales contain few items (Nunnally and Bernstein 1994).

Floor and ceiling effects were examined by estimating the percentage of participants obtaining the lowest and highest possible scores on the GPTS‐8 subscales and total score (Terwee et al. 2007).

The factorial structure of the GPTS‐8 was examined using confirmatory factor analysis (CFA). CFA tested one‐, two‐, bifactor, and second‐order models of the GPTS‐8. As Mardia's test (Mardia 1970) indicated non‐normality (Mardia skewness = 3956.7, *p* < 0.0001; kurtosis = 93.65, *p* < 0.0001) and the data were ordinal, analyses employed maximum likelihood estimation with robust (Huber–White) standard errors and a scaled test statistic (MLR). Model fit was evaluated using χ^2^, the Comparative Fit Index (CFI), the Root Mean Square Error of Approximation (RMSEA), and the Standardized Root Mean Square Residual (SRMR). Acceptable model fit was defined as RMSEA ≤ 0.08, SRMR ≤ 0.09, and CFI ≥ 0.90, acknowledging that χ^2^ is sensitive to sample size (Hu and Bentler 1999). Internal consistency was further assessed using McDonald's ω, which provides a more accurate estimate of true reliability (McDonald 1999).

To evaluate the unidimensionality of the general factor derived from the bifactor model, the explained common variance (ECV), percentage of uncontaminated correlations (PUC), and Omega Hierarchical (ωH) were computed (Rodriguez et al. 2016a). Approximate unidimensionality was assumed when ECV > 0.60 and either ωH > 0.70 or PUC > 0.70 (Rodriguez et al. 2016a). We also reported factor determinacy (FD) to assess the reliability of estimated factor scores and their correspondence with the underlying latent constructs (Gorsuch 1983; Muthén and Muthén 2017), and the Item Explained Common Variance (IECV) to evaluate the proportion of each item's common variance accounted for by the general factor, thus indicating item‐level unidimensionality (Stucky and Edelen 2015). To aid interpretation of the general latent dimension, correlations between individual item scores and the latent factor (θ) were computed as indicators of item–factor association, reflecting the degree to which each item represented the underlying construct (Brown 2015). CFA models were estimated using the *lavaan* package in *R* (Rosseel 2012), and bifactor indices were computed with the *BifactorIndicesCalculator* package (Dueber 2021).

Convergent validity was examined against the PQ‐B, using the at‐risk subgroup as a referent term. Participants classified as above versus below the psychosis‐risk threshold were compared using Welch's *t*‐tests on the two GPTS‐8 subscales and the global score (paranoia factor).

## 3 Results

Cronbach's alpha indicated good reliability for the PQ‐B frequency (0.83) and distress (0.85) scores. Reliability was acceptable for the GPTS‐8 total score (0.78) and its subscales (Ideas of Reference = 0.68; Ideas of Persecution = 0.66). Participants used the full 1–5 response range for all items except item 1 (“I spent time thinking about friends gossiping about me”). While some items exceeded conventional thresholds for skewness and kurtosis, the overall distributions were considered adequate for the planned analyses using robust estimation methods (Table 1).

**TABLE 1:** Brief version of the “Green et al. Paranoid Thoughts Scale”.

	Item	Mean (SD)	Observed range	Skewness	Kurtosis	IECV	r with *θ*
1.	I spent time thinking about friends gossiping about me. (IR) Ho trascorso del tempo a pensare ad amici che spettegolano su di me.	1.2 (0.5)	1 to 3	2.3	4.7	0.46	0.47
2.	I often heard people referring to me. (IR) Ho sentito spesso la gente fare allusioni su di me.	1.5 (0.8)	1 to 5	1.8	2.8	0.99	0.78
3.	I have been upset by friends and colleagues judging me critically. (IR) Sono stato turbato a causa di amici o colleghi che mi criticavano.	1.6 (0.9)	1 to 5	1.4	1.4	0.85	0.64
4.	I was frustrated by people laughing at me. (IR) Ero frustrato dalla gente che rideva di me.	1.2 (0.6)	1 to 5	3.9	18.6	0.49	0.62
5.	I was sure someone wanted to hurt me. (IP) Ero sicuro che qualcuno volesse farmi del male.	1.1 (0.6)	1 to 5	4.8	24.8	0.42	0.44
6.	I was distressed by being persecuted. (IP) Ho provato disagio a causa delle persecuzioni che subivo.	1.1 (0.5)	1 to 5	4.6	23.9	0.93	0.72
7.	It was difficult to stop thinking about people wanting to make me feel bad. (IP) Era difficile smettere di pensare alle persone che volevano farmi sentire male.	1.2 (0.7)	1 to 5	3.0	9.3	0.31	0.64
8.	People have been hostile toward me on purpose. (IP) Certa gente mi è stata ostile di proposito.	1.6 (1.0)	1 to 5	1.6	1.5	0.99	0.69
	Ideas of reference	5.5 (2.1)	4 to 16	1.9	4.7		
	Ideas of persecution	5.1 (2.0)	4 to 15	2.0	4.0		
	GPTS‐8 (“paranoia”)	10.1 (3.6)	8 to 27	1.9	3.6		

*Note:* The item's content is presented in both English and Italian. Items are scored 1–5; subscales range 4–16; total GPTS‐8 ranges 8–32. in the bifactor model, proportion of each item's shared variance explained by the general factor, controlling for specific factors; *r* = Pearson correlation between each item score and the latent factor score (θ) for the global *Paranoia* dimension, indicating how closely each item reflects the underlying latent construct.

Abbreviations: IECV: Item‐level Explained Common Variance; IP: Ideas of persecution; IR: Ideas of reference.

### 3.1 Floor and Ceiling Effects

Floor effects were observed for both subscales (46.5% for Ideas of Reference and 66.7% for Ideas of Persecution) and for the GPTS‐8 total score (40.3%), while no participants obtained the maximum possible score on any scale, indicating the absence of ceiling effects.

### 3.2 Gender and Age Distribution of the Scores

Based on the summed‐score structure of the scale, no significant gender differences emerged for the GPTS‐8 total or subscale scores (all *p* > 0.40). Higher GPTS‐8 scores were associated with younger age for the total score (*r* = −0.157, *p* = 0.014) and the Ideas of Reference subscale (*r* = −0.158, *p* = 0.014). The association with the Ideas of Persecution subscale was in the same direction but did not reach statistical significance (*r* = −0.120, *p* = 0.061).

### 3.3 Results of the CFA

Among tested CFA models, the bifactor solution showed comparatively better fit (see Table 2).

**TABLE 2:** Confirmatory factor analysis of the GPTS‐8 in the tested models. Robust fit indexes.

Model	χ^2^	df	P	CFI	RMSEA (90%CI)	SRMR	Cronbach's *α*	McDonald's *ω*	AIC	BIC
Unidimensional	52.2	20	< 0.001	0.859	0.115 (0.077–0.153)	0.072	PAR = 0.782	PAR = 0.791	3579	3635
Correlated two‐factor	44.2	19	< 0.01	0.907	0.096 (0.059–0.133)	0.066	IR = 0.684 IP = 0.660	IR = 0.703 IP = 0.686	3552	3611
Second‐order two factor model	41.8	18	< 0.01	0.907	0.098 (0.059–0.137)	0.066	IR = 0.684 IP = 0.660	IR = 0.703 IP = 0.686 PAR = 0.769	3554	3617
Bifactor	17.4	12	0.136	0.983	0.052 (0.00–0.102)	0.032	IR = 0.684 IP = 0.660 PAR = 0.781	IR = 0.338 IP = 0.425 PAR = 0.808	3517	3601
Threshold for fit			*p* > 0.05	≥ 0.90	< 0.08	< 0.09	≥ 0.70	≥ 0.80	↓↓	↓↓

Abbreviations: IP: Ideas of persecution; IR: Ideas of reference; PAR = Paranoia.

Despite a Percent of Uncontaminated Correlations (PUC = 0.57) below the > 0.70 benchmark, the Explained Common Variance (ECV = 0.63) and omega hierarchical (*ω*_h_ = 0.70) indicated acceptable dominance of the general factor. Factor determinacy values suggested adequate representation of the global Paranoia factor (0.85) and the Ideas of Persecution subscale (0.93), but lower representation for the Ideas of Reference subscale (0.66). This pattern indicates that most shared variance is captured by the general factor, with the specific reference dimension being comparatively under‐defined, a configuration frequently observed in bifactor models (Reise 2012; Rodriguez et al. 2016a, 2016b).

Item Explained Common Variance (IECV) coefficients (Table 1) showed that items 2, 3, 6, and 8 primarily reflected the general *Paranoia* factor (IECV ≥ 0.85). Items 1, 4, and five exhibited mixed contributions from the general and specific dimensions (IECV ≈ 0.42–0.50), whereas item 7 mainly captured variance specific to *Ideas of persecution* (IECV = 0.32). Consistent with this, item 7 loaded strongly on the *Ideas of persecution* subscale (0.82) and moderately on the global *Paranoia* factor (0.56; Figure 1).

**FIGURE 1:**
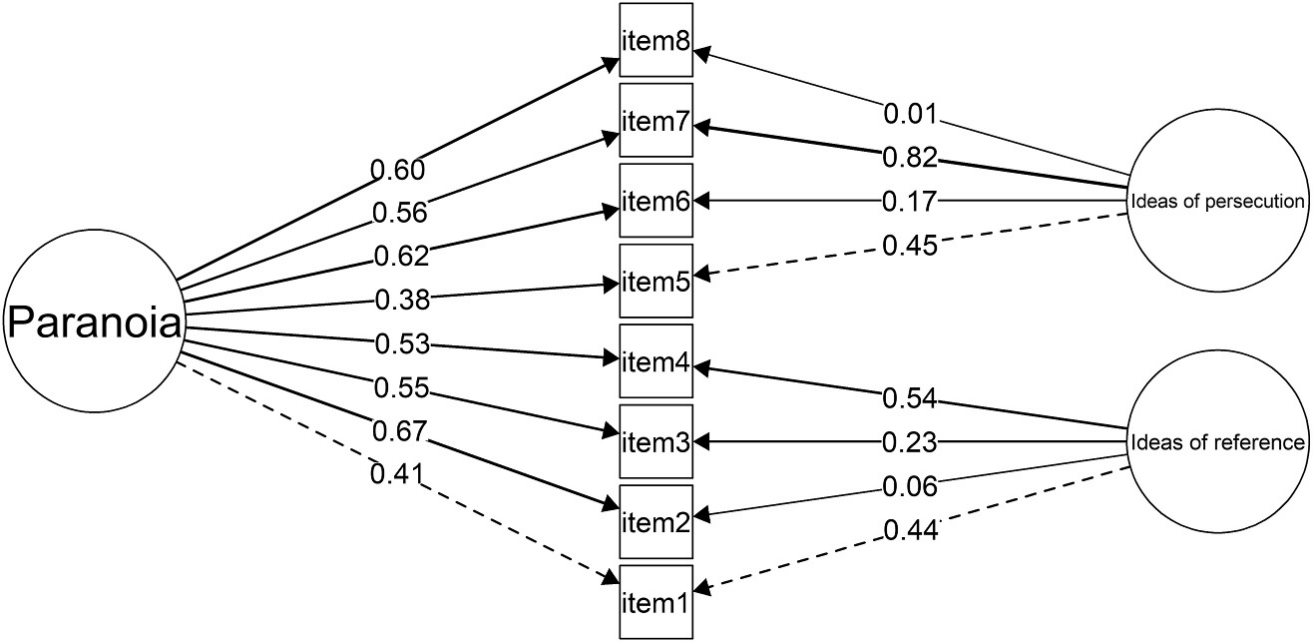
Confirmatory factor analysis of the Italian short version of the *Green* et al. Paranoid Thoughts Scale (GPTS‐8). The diagram depicts the bifactor model with two specific factors, showing item loadings on the global *Paranoia* factor as well as on the two specific components, Ideas of Reference and Ideas of Persecution.

Correlations between individual item scores and the latent *Paranoia* factor (θ) ranged from 0.44 to 0.78, indicating variable alignment with the global construct (Table 1). Items 2, 6, and 8 showed the strongest associations (*r* ≥ 0.70), suggesting that they are highly representative of the general paranoia dimension. Items 3, 4, and 7 displayed moderate correlations (*r* ≈ 0.62–0.64), while items 1 and 5 showed weaker associations (*r* < 0.50), reflecting greater specificity or residual variance. Most GPTS‐8 items showed limited variability at lower levels of latent paranoia but increased more sharply at higher *θ* values, indicating greater differentiation among individuals with elevated paranoid ideation (Figure 1A in supplementary material).

Overall, the observed pattern of loadings, IECV coefficients, and item–factor correlations was consistent with a bifactor structure in which items 2, 3, 6, and 8 primarily reflect the general Paranoia factor.

### 3.4 Convergent Validity

The at‐risk subgroup on the PQ‐B scored significantly higher on both GPTS‐8 subscales and the global *Paranoia* factor, with large to very large effect sizes (Supporting Information S1).

## 4 Discussion

The Italian version of the GPTS‐8—a brief adaptation of the original GPTS and R‐GPTS—demonstrated satisfactory reliability, factorial validity, and convergent validity in a non‐clinical sample. The scale captured the core dimensions of paranoid ideation while providing a concise and efficient assessment suitable for large‐scale research applications. Reliability was generally acceptable. McDonald's omega exceeded Cronbach's alpha, which is reported primarily for comparability with previous studies. In the bifactor model, however, omega values for the specific factors were substantially reduced after accounting for variance attributable to the general paranoia factor, suggesting that the most reliable variance was captured by the overarching construct.

Marked floor effects were observed across subscales. Although this pattern is consistent with the expected distribution of paranoid ideation in non‐clinical student populations, it has important measurement implications. Strong floor effects indicate limited variability at the lower end of the latent trait and may reduce the instrument's ability to discriminate among individuals reporting minimal or absent paranoid ideation. Conversely, the GPTS‐8 appeared more sensitive at higher levels of the construct, suggesting that it is better suited to identifying elevated paranoia than to detecting subtle differences at the lowest end of the continuum. The restricted response range observed for Item 1 may similarly reflect characteristics of the sample, as suspiciousness‐related experiences are likely to be infrequent in non‐clinical populations. Moreover, concerns about friends gossiping may be interpreted as relatively common social worries rather than distinctly paranoid experiences, discouraging endorsement of the highest response categories. To our knowledge, no previous study has formally examined floor or ceiling effects in the GPTS‐8. The present findings, therefore, provide novel information regarding the instrument's operating range and suggest that future research should evaluate its performance in more diverse and clinically enriched samples.

Consistent with the original validation study (Bianchi and Verkuilen 2021), bifactor modeling supported a dominant general paranoia factor alongside more specific reference and persecution dimensions. Item‐level diagnostics indicated that several items loaded primarily on the general factor, supporting contemporary dimensional models in which a broad latent paranoia dimension accounts for most shared variance across paranoid experiences (Freeman et al. 2021; Schlier et al. 2024). From a psychometric perspective, these findings suggest that the GPTS‐8 functions predominantly as a measure of global paranoia and support the use of the total score as a defensible summary index in research settings.

At the same time, the reference factor showed lower factor determinacy and more heterogeneous item contributions than the persecutory factor, indicating that ideas of reference were represented less consistently than persecutory beliefs. Several non‐mutually exclusive explanations may account for this finding. First, the strong general factor may reflect the tendency for paranoid ideation in non‐clinical populations to manifest as a broad dispositional construct rather than as clearly differentiated symptom dimensions. Second, ideas of reference may be particularly sensitive to sociocultural and interpersonal context, requiring respondents to interpret ambiguous social cues and everyday interactions that are shaped by cultural norms and expectations regarding social evaluation. This interpretation is consistent with evidence suggesting greater cross‐cultural variability in reference‐related experiences than in persecutory beliefs (Schlier et al. 2024). Third, the brevity of the GPTS‐8 may limit the reliable variance available to distinguish between closely related dimensions. Because each domain is represented by only four items, the predominance of the general factor may partly reflect the psychometric consequences of short‐form measurement in addition to the underlying structure of paranoia itself. Future studies comparing the GPTS‐8 with longer versions of the scale and examining its performance across clinical and cultural contexts will help clarify the relative contribution of substantive and measurement‐related factors to the observed structure.

Although formal measurement invariance could not be evaluated due to sample‐size limitations and the complexity of bifactor models, the replication of the general factor structure across studies provides preliminary support for the GPTS‐8 as a potentially useful instrument for cross‐cultural research and international data harmonization.

Finally, convergent validity with the PQ‐B was consistent with theoretical expectations, indicating that higher GPTS‐8 scores were associated with greater endorsement of psychotic‐like experiences. This association should be interpreted dimensionally, as the two instruments assess overlapping but non‐identical constructs. Together with the replication of the scale's latent structure, these findings extend previous validations by providing the first Italian adaptation of the GPTS‐8 and further support its applicability across cultural contexts.

Overall, the findings support the GPTS‐8 as a brief and psychometrically sound measure for epidemiological studies, cross‐cultural investigations, and research adopting dimensional models of paranoia. Future work should examine measurement invariance across demographic and clinical groups, as well as longitudinal stability and predictive validity in clinical and high‐risk populations.

### 4.1 Strengths and Limitations

The study's strengths include the use of rigorous analytic methods, including bifactor modeling and item‐level diagnostics, the adoption of a well‐characterized psychosis‐risk comparator (PQ‐B), and the contribution of the first Italian short‐form measure of paranoia suitable for large‐scale epidemiological or digital research, pending validation in representative samples. Several limitations should also be acknowledged. The sample consisted exclusively of university students, representing a relatively homogeneous non‐clinical population and limiting generalizability to the broader Italian population. In addition, the cross‐sectional design precludes examination of temporal stability, and the sample size did not permit formal testing of measurement invariance across subgroups. While these constraints limit the scope of generalization, the study was designed to establish the structural and distributional properties of the GPTS‐8 within a non‐clinical context prior to extension into clinical samples. Establishing these foundational psychometric properties represents a necessary step before more demanding evaluations—such as measurement invariance, clinical validation, or longitudinal stability—can be meaningfully addressed.

## 5 Conclusion

The present findings provide initial support for the validity of the Italian GPTS‐8 and extend the availability of this brief measure of paranoid ideation to a major European language. The findings support its use in non‐clinical Italian populations and add to the growing body of evidence for the cross‐cultural applicability of the GPTS‐8.

The generalizability of these findings is limited by the homogeneous non‐clinical sample and the absence of formal measurement invariance testing. Future research should evaluate the instrument in more diverse and clinically enriched populations, examine longitudinal stability, and test measurement invariance across cultural and demographic groups.

## Author Contributions

**Antonio Preti:** conceptualization, methodology, formal analysis, visualization, data curation, writing – original draft. **Carmelo Masala:** resources, supervision, project administration, writing – review and editing. **Donatella Rita Petretto:** conceptualization, methodology, investigation, resources, data curation, project administration, writing – review and editing.

## Funding

The authors have nothing to report.

## Ethics Statement

The authors certify that all procedures performed in this study were conducted in accordance with the ethical standards of the relevant institutional review boards and with the principles outlined in the Declaration of Helsinki (1975) and its subsequent amendments.

## Conflicts of Interest

The authors declare no conflicts of interest.

## Supporting information


Supporting Information S1

## Data Availability

The data that support the findings of this study are available on request from the corresponding author. The data are not publicly available due to privacy or ethical restrictions.
