# Supplementary material for: Psychometric Validation of the Italian 8‐Item Green Paranoid Thoughts Scale (GPTS‐8)
Source: Int J Methods Psychiatr Res. 2026 Sep 30;35(4):e70097. doi: 10.1002/mpr.70097 (PMC13627870; doi:10.1002/mpr.70097)
Supplement: Supplementary file 1 [file MPR-35-e70097-s001.docx]

Appendix – Supplementary materials

Table 1A. Concurrent validity of the GPTS-8 total score and its subscales.

Values are mean (standard deviation)

|  | PQ-B below cut-off  n.=198 | PQ-B above cut-off  n.=45 | Welch’s t-test | Hedges’s g |
| --- | --- | --- | --- | --- |
| *Ideas of reference* | 5.1 (1.6) | 7.1 (3.0) | t(49.9)=-4.2; p<0.001 | 0.99 |
| *Ideas of persecution* | 4.8 (1.6) | 6.6 (2.8) | t(50.8)=-4.3; p<0.001 | 0.98 |
| *Paranoia* | 9.9 (2.8) | 13.7 (5.1) | t(49.9)=-4.8; p<0.001 | 1.14 |

Note: Subscale and total scores of the GPTS-8 were computed as simple sums of the corresponding items, providing a practical estimate of individual standing on the scale. These scores should be interpreted with caution, as the bifactor model indicated that some items reflect specific variance beyond the general *Paranoia* factor.

Because Levene’s test indicated unequal variances, group comparisons were conducted using Welch’s *t*-test, which applies adjusted degrees of freedom.

Figure 1A. Relationship between item responses and the latent *Paranoia* factor (θ). Points show observed scores; blue lines represent LOESS (locally estimated scatterplot smoothing) fits, illustrating nonlinear associations. Most items display limited variability at low θ and steeper increases at higher θ. Panel annotations report Pearson correlations (*r*) between each item and θ.
